# Enteropathogenic *Klebsiella pneumoniae* HIV-Infected Adults, Africa

**DOI:** 10.3201/eid0901.020138

**Published:** 2003-01

**Authors:** Phuong L. Nguyen Thi, Simon Yassibanda, Awa Aidara, Chantal Le Bouguénec, Yves Germani

**Affiliations:** * Institut Pasteur de Bangui, Bangui, Central African Republic; † Hôpital de l'Amitié, Bangui, Bangui, Central African Republic; ‡ Institut Pasteur de Dakar, Dakar, Sénégal; § Institut Pasteur, Paris, France

**Keywords:** AIDS, chronic diarrhea, *Klebsiella pneumonia*, *letter*

**To the Editor**: Although *Klebsiella pneumoniae* lives as a commensal in the intestine, this bacterium can occasionally cause diarrhea in HIV-negative persons (*1*–*4*). Some of these diarrheagenic strains encode thermostable or thermolabile toxins (*2*). One group of researchers showed that a *K. pneumoniae* strain isolated from bloody diarrhea can bind to HeLa cells and cytoskeletal proteins, such as the actin that accumulates at the point of bacterium-host contact (*3*). However, this isolate did not contain any of the genes encoding virulence factors that have been ascribed to pathogenic *Escherichia* *coli* strains and are responsible for bloody diarrhea or dysenteric syndromes (*3*).

In Bangui, the rate of isolation of pure cultures of *K. pneumoniae* from the stools of immunocompromised HIV-infected adults with chronic diarrhea is increasing. This finding was observed during the routine biological analyses performed in the Pasteur Institute Medical Laboratory and is consistent of that made by Gassama et al. in Dakar (*5*). The role of *K. pneumoniae* in HIV-infected adults is not well documented. As no other known enteric pathogens were isolated from these samples, we conducted a case-control study in Bangui in 1999–2001 to determine the clinical significance of *K. pneumoniae*. The study population included 31 adults hospitalized with chronic diarrhea and 31 matched controls. (Because of civil unrest in Bangui due to military rebellions and the difficulties involved in recruiting controls, the study was performed on a small sample.) To be included in the study, the patients had to be HIV positive, be >18 years of age, have provided a stool sample containing *K. pneumoniae* on the day of recruitment, and have given informed consent. Inclusion criteria were the same for controls except that they did not have diarrhea on the day of recruitment or in the previous month. Controls were family members or neighbors of the patients, matched by age (within 5 years) and sex. Specimens from cases and controls were collected over the same 1-month period. Known enteric pathogens were identified by standard methods as described (*6*). Endoscopic examinations were used to diagnose pseudomembranous colitis in patients with bloody chronic diarrhea or watery chronic diarrhea. The median CD4+ cell count was 122 cells/μL in the patients and 436 cells/μL in the controls. AIDS-related symptoms were observed in all of the cases (Centers for Disease Control and Prevention [CDC] stage C2 or C3) and none of the controls (CDC stage A1). Of the 31 patients, 7 (22.6%) had bloody chronic diarrhea, 9 (29%) had watery chronic diarrhea, and 15 (48.4%) had mild chronic diarrhea. Pseudomembranous colitis was diagnosed in nine patients (six with bloody diarrhea, three with watery chronic diarrhea) who had been taking several antibiotics, including ampicillin, for a long period (>1 month). Five *K. pneumoniae* colonies were randomly picked up from each case sample and examined. Control colonies were chosen if their appearance suggested *K. pneumoniae*. The mean number of strains tested was 4.99 for patients and 4.64 for controls (not significant, p=0.969). All of the enteric bacteria isolated from the patients and grown on nonselective bromocresol purple medium were *K. pneumoniae,* whereas an average of 10% to 20% of the enteric bacteria isolated from the controls were *K. pneumoniae*. We used assays typically used to identify the virulence factors of diarrheagenic *E. coli* (*7*) to characterize the virulence properties of the *K. pneumoniae* isolates, their genotypes, and their phenotypes (their ability to bind to cultured HEp-2 cells and to promote cytoskeleton modifications [fluorescent actin staining test], to invade epithelial cells, to produce various enterotoxins and cytotoxins, and to induce fluid accumulation in the intestines of newborn mice). The rabbit ligated ileal loops test was performed when the genetic or phenotypic (on Vero or Y1 cells) tests were positive for toxins. All isolates from 27 patients (7, 9, and 11 with bloody, watery, and mild chronic diarrhea, respectively) and two of the isolates from one control displayed an aggregative adherence phenotype on HEp-2 cells. This phenotype appeared to be significantly associated with chronic diarrhea (27/31 cases vs. 1/31 controls, χ =40.7, p<10^-6^). All HEp-2–adherent *K. pneumoniae* isolated from six of the patients produced toxins. The culture supernatants of the HEp-2–adherent *K. pneumoniae* strains isolated from four of the patients with bloody chronic diarrhea and pseudomembranous colitis had cytotoxic effects on Vero and Y1 cells, as characterized by the rounding of cells after 24 h, followed by their detachment from the culture plate and death after 72 h. These effects were not neutralized by rabbit antisera raised against the Shiga toxin or the cholera toxin. The HEp-2–adherent *K. pneumoniae* strains isolated from two patients with watery chronic diarrhea were enterotoxigenic in ligated rabbit ileal loops. Only the HEp-2–adherent *K. pneumoniae* strains isolated from one patient with watery chronic diarrhea and pseudomembranous colitis (5 strains) and from four patients with mild chronic diarrhea (20 strains) carried sequences related to virulence genes from pathogenic enteroaggregative *E. coli*. These isolates were all positive for the *astA* gene, which encodes the EAST1 toxin, and for the genes that produce the AAF/I fimbriae.

*K. pneumoniae* is normally resistant to β-lactams. Multidrug-resistant *K. pneumoniae* have been reported (*1*). All *K. pneumoniae* isolates in this study were resistant to several antibiotics including cotrimoxazole and ampicillin, which are largely used in Bangui according to the recommendations of the World Health Organization (*8*). In addition to the nonspecific measures used to correct and prevent fluid, electrolyte, and nutritional imbalances, all persons with bloody and watery chronic diarrhea (including those with pseudomembranous colitis) and 5 of the 15 patients with mild chronic diarrhea (10 were lost to follow-up) were treated with ofloxacin (800 mg/day) or ceftriaxone (2 g/day), based on the results of antimicrobial susceptibility testing. The state of all patients with pseudomembranous colitis and mild chronic diarrhea, and of five of the patients with watery chronic diarrhea (one patient died), improved within 10 days of treatment.

In Dakar, during the study describing ordinary and opportunistic enteropathogens associated with diarrhea in adults (*5*), stool samples were collected from five HIV-infected adults with watery chronic diarrhea. In all cases, heavy *K. pneumoniae* growth was observed on the primary culture media, and no other known pathogens were recovered. These *K. pneumoniae* strains were subjected to the same phenotypic and genotypic tests as the strains isolated in Bangui. HEp-2–adherent *K. pneumoniae* was identified in four of these five samples. The condition of all the patients rapidly improved after treatment with ofloxacin. In Bangui and Dakar, repeated stool cultures were negative for *K. pneumoniae* by the end of treatment, providing further evidence that these *K.* *pneumoniae* were of etiologic importance, especially the HEp-2–adherent *K. pneumoniae* strains.

Only seven patients (four with mild, two with watery, and one with bloody chronic diarrhea) had not taken antibiotics during the 2 weeks before stool collection. The stool specimens from these seven patients yielded pure primary cultures of HEp-2–adherent *K. pneumoniae* and no other bacterial enteric pathogens. None of these seven participants was diagnosed with pseudomembranous colitis. The HEp-2–adherent *K. pneumoniae* strains isolated from the two participants with watery chronic diarrhea induced the accumulation of fluid in ligated rabbit ileal loops, and the HEp-2–adherent strains isolated from three of the participants with mild chronic diarrhea carried the *astA* gene, which is associated with pathogenic EAEC. Among the five patients with pseudomembranous colitis, all of whom had received antibiotics before the onset of illness, we found that the four isolates from the patients with bloody chronic diarrhea were cytopathogenic; the one isolate from the patient with watery chronic diarrhea had the pathogenic marker for enteroaggregative *E. coli*. These findings suggest that not only is *K. pneumoniae* associated with chronic diarrhea in HIV-infected persons but also that infection with particular HEp-2-adherent *K. pneumoniae* subtypes may be associated with specific clinical illness.
